# Amylases: Biofilm Inducer or Biofilm Inhibitor?

**DOI:** 10.3389/fcimb.2021.660048

**Published:** 2021-04-27

**Authors:** Dibyajit Lahiri, Moupriya Nag, Ritwik Banerjee, Dipro Mukherjee, Sayantani Garai, Tanmay Sarkar, Ankita Dey, Hassan I. Sheikh, Sushil Kumar Pathak, Hisham Atan Edinur, Siddhartha Pati, Rina Rani Ray

**Affiliations:** ^1^ Department of Biotechnology, University of Engineering & Management, Kolkata, India; ^2^ Department of Food Technology and Bio-Chemical Engineering, Jadavpur University, Kolkata, India; ^3^ Malda Polytechnic, West Bengal State Council of Technical Education, Government of West Bengal, Malda, India; ^4^ Department of Biotechnology, Maulana Abul Kalam Azad University of Technology, Haringhata, India; ^5^ Faculty of Fisheries and Food Science, Universiti Malaysia Terengganu, Kuala Nerus, Malaysia; ^6^ Department of Bioscience and Bioinformatics, Khallikote University, Berhampur, India; ^7^ School of Health Sciences, Universiti Sains Malaysia, Penang, Malaysia; ^8^ Centre of Excellence, Khallikote University, Berhampur, India; ^9^ Research Division, Association for Biodiversity Conservation and Research (ABC), Balasore, India

**Keywords:** biofilm, sessile, antibiofilm, amylase, antimicrobial

## Abstract

Biofilm is a syntrophic association of sessile groups of microbial cells that adhere to biotic and abiotic surfaces with the help of pili and extracellular polymeric substances (EPS). EPSs also prevent penetration of antimicrobials/antibiotics into the sessile groups of cells. Hence, methods and agents to avoid or remove biofilms are urgently needed. Enzymes play important roles in the removal of biofilm in natural environments and may be promising agents for this purpose. As the major component of the EPS is polysaccharide, amylase has inhibited EPS by preventing the adherence of the microbial cells, thus making amylase a suitable antimicrobial agent. On the other hand, salivary amylase binds to amylase-binding protein of plaque-forming *Streptococci* and initiates the formation of biofilm. This review investigates the contradictory actions and microbe-associated genes of amylases, with emphasis on their structural and functional characteristics.

## Introduction

Biofilm is a consortium of sessile microbial species formed on the surfaces of various natural habitats. Eighty per cent of infections may be caused by biofilm-associated pathogens (Donlan and Costerton, 2002; Lahiri et al., 2021b), and about 90% of the mass of biofilm is composed of extracellular polymeric substance (EPS) (Costerton, 1999). The biofilm stores carbohydrates, proteins, and nucleic acids, which provide nutrients to the developing sessile communities and stabilize indwelling cells. This action also mediates attachment of the sessile cells to the biotic or abiotic surfaces and acts as a scaffold for the enzymes and cells and the attachment of antibiotics (Stewart and Costerton, 2001; Flemming and Wingender, 2010; Mann and Wozniak, 2012).The EPS associated with the biofilm consist of various types of cationic and anionic molecules, such as glycoproteins, glycolipids, and proteins, that can bound with antimicrobial agents, thus providing shelter for microbial species (Nadell et al., 2015). The EPS acts as a coating to protect bacterial cells from antibiotics, thereby enhancing tolerance of the bacteria to the drug. Most of the biofilm matrix consists of extracellular polysaccharides that crosslink with eDNA, thereby stabilizing the structure of the biofilm. The development of resistance to drugs is mainly due to the presence of e-DNA since it can be easily absorbed onto the bacteria, thereby promoting DNA communication (Madsen et al., 2012).

The structural components of the EPS play important roles in the development of the biofilm. DNABII is a structural protein that helps stabilize the biofilm (Devaraj et al., 2015), which is followed by activation of the quorum sensing (QS) pathway, thereby facilitating the development of biofilm (Rasamiravaka et al., 2015). The sessile microcolonies masked within the matrix are concerning, as, compared with planktonic form (Lam et al., 2015), they decrease the permeability of bactericides to enhance drug tolerance. The development of resistance can be due to the rapid exchange of DNA, thereby rendering the antibiotic ineffective by decreasing its antibacterial property (Król et al., 2013; Jennings et al., 2015). The development of resistance reduces the effectiveness of traditional treatments regarding the biofilm, which is a serious concern among health practitioners. The development of EPS results in changes that lead to physiological drift and the development of special environments of oxygen gradient and local acidity (Chang et al., 2015).

**Figure 1:**
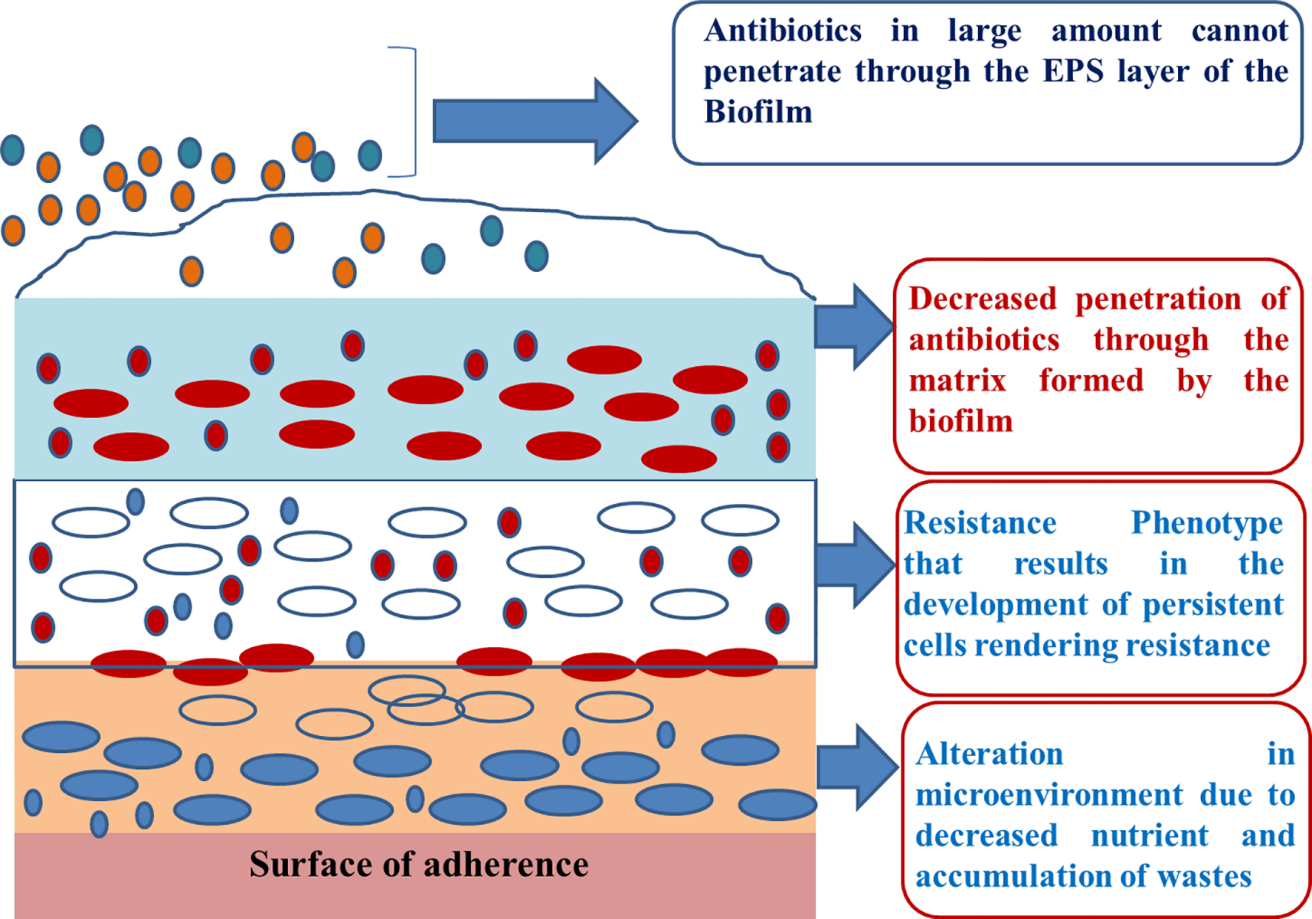
Development of resistance against antimicrobial substances.

The EPS also prevents the penetration of drug molecules to the sessile cells, thus resulting in the development of antimicrobial resistance (**Figure 1**). Bacterial resistance against various antimicrobial agents, including antibiotics, is an emerging health care crisis (Jana et al., 2017) and has significantly affected the global economy. Most chronic bacterial infections are linked to the development of biofilms, and in-dwelling bacterial colonies are inherently resistance to host immune responses (Patel et al., 2014).

Hypoxia imparts tolerance in the biofilm towards antimicrobials; biofilms exposed to antimicrobials in anaerobic environments were more resistant than were those exposed in aerobic environments (Borriello et al., 2004). Accumulation of colistin-resistant subpopulations within the biofilm determines the toxic region within the biofilm, which in turn indicates the decreased growth of the biofilm and enhanced tolerance towards antimicrobials. Hypoxia reduces the potential of the outer membrane of the bacterial cell, resulting in the development of antibiotic resistance against aminoglycosides. **Table 1** lists the genes associated with the development of bacterial resistance.

**Table 1:** Genes responsible for the development of resistance within bacterial cells.

Gene(s)	Antibiotics	Gene product(s)	Proposed mechanism of protection	References
*brlR*	Tobramycin, norfloxacin, trimethoprim, tetracycline, kanamycin, chloramphenicol	Transcriptional regulator	Upregulation of multidrug efflux pumps	(Spoering and Lewis, 2001)
*sagS*	Tobramycin, norfloxacin	Two-component hybrid	Activation of BrlR by promoting increased c-di-GMP levels	(Webb et al., 2003)
*ndvB*	Tobramycin, gentamicin, ciprofloxacin	Glucosyltransferase	Sequestration of antibiotics, upregulation of ethanol oxidation genes	(Thomas et al., 2009)
*exaA, pqqC, erbR*	Tobramycin	Ethanol oxidation players	Unknown	(Kohanski et al., 2010)
*PA1875-1877*	Tobramycin, gentamicin, ciprofloxacin	Biofilm-specific antibiotic efflux pump	Efflux of antibiotics out of the cell	(Zheng and Stewart, 2004)
*tssC1, hcp1*	Tobramycin, gentamicin, ciprofloxacin	Type VI secretion components	Unknown	(Webb et al., 2003)
*PA0756-0757*	Tobramycin, gentamicin	Two-component system	Unknown	(Kohanski et al., 2010)
*PA2070*	Tobramycin, gentamicin	TonB-dependent receptor	Unknown	(Lechner et al., 2012)
*PA5033*	Tobramycin, gentamicin	Hypothetical proteins	Unknown	(Webb et al., 2003)
*pslABCDEFGHIJKLMNO*	Colistin, polymyxin B, tobramycin, ciprofloxacin	Psl biosynthetic enzymes	Unknown	(Zheng and Stewart, 2004)
*pelABCDEFG*	Tobramycin, gentamicin	Pel biosynthetic enzymes	Unknown	(Webb et al., 2003)
*relA, spoT*	Ofloxacin, meropenem, colistin, gentamicin	Players in the stringent response	Upregulate antioxidant defenses and downregulate pro-oxidants	(Zheng and Stewart, 2004)
*rapA*	Penicillin G, norfloxacin, chloramphenicol, gentamicin	Helicase-like protein	Upregulation of YhcQ and of exopolysaccharide synthesis	(Van Acker and Coenye, 2016)
*yafQ*	Tobramycin, cefazolin	Toxin	Persister cell formation	(Kohanski et al., 2010)
*epaOX*	Gentamicin	Glycolsyltranferase	Maintenance of cell wall integrity	(Whiteley et al., 2001)
*epaI*	Daptomycin	Glycolsyltranferase	Unknown	(Whiteley et al., 2001)
*gelE*	Gentamicin, daptomycin, linezolid	Gelatinase	Unknown	(Spoering and Lewis, 2001)
*fsrA, fsrC*	Gentamicin, daptomycin, linezolid	Quorum-sensing players	Unknown	(Thomas et al., 2009)
*dltABCD*	Gentamicin	Enzymes involved in D-alanylation of teichoic acid	Decrease in the negative charge of the cell wall	(Van Acker and Coenye, 2016)

Resistance to antimicrobial drugs is mediated by EPS, which renders conventional drugs ineffective. This effect has led to the drift from conventional methods of treatment to the use of other agents, such as plant secondary metabolites, antimicrobial peptides, and enzymes, as therapeutic measures (Schachtele et al., 1975; Juntarachot et al., 2020). Enzymes have been effective anti-biofilm agents, and they are environmentally friendly and easily biodegradable (Xavier et al., 2005). Enzymes can inhibit biofilms when the bacterial exopolysaccharides serve as substrate (Brisou, 1995; Sutherland, 1995). Application of suitable enzymes for degrading the structural components of the biofilm matrix will weaken it so that it can be more easily removed by mechanical processes. Since the sugar backbone of the biofilm matrix is composed mainly of carbohydrate residues, carbohydrate-based enzymes, such as amylase, might be used to hydrolyze and thereby denature the biofilm matrix (Lembre et al., 2012).

On the other hand, oral biofilm contains amylase binding proteins, which may indicate that that amylases play a role in establishing the biofilm (Rogers et al., 2001). For example, α-amylase in human saliva binds to α-amylase-binding proteins (ABPs) that are present on bacterial surfaces. Glucose and maltose released from processed starches by salivary amylase are metabolized by oral bacteria to form the biofilm of dental plaque. This process induces oral colonization by streptococci, which leads to the formation of oral biofilm by the plaque-forming bacteria (Nikitkova et al., 2013) The extracellular protein network of AbpA-amylase-Gtf may influence the ecology of oral biofilms, likely during initial phases of colonization. Thus, AbpA-amylase-Gtf may help in co-aggregation and colonization within the oral cavity. The functional significance of amylase binding proteins in oral colonization by *Streptococci* is important for understanding how salivary components influence oral biofilm formation by these important dental-plaque species. Therefore, the question is raised of whether amylase assists in the formation of dental biofilm or, paradoxically, it can be used as a biofilm inhibitor (Haase et al., 2017; Wu et al., 2020). Amylase seems to be useful for removing biofilm by disintegrating the carbohydrate moiety of the biofilm matrices, but at the same time it can also induce biofilm formation. The present review explores the evidence on the role of amylase in biofilm formation at the molecular level and the mechanisms that may use for eradicating the biofilm.

## Background of Biofilm Formation

A biofilm is an assemblage of microbial cells that is irreversibly associated with a surface and is enclosed in a matrix mostly made of polysaccharide material (Donlan and Costerton, 2002). The lower layers of a biofilm contain microbes that are bound together in a polysaccharide matrix with other organic components such as eDNA, proteins, and inorganic materials. The upper layer is a loose amorphous layer extending into the surrounding medium. The fluid layer bordering the biofilm has stationary and dynamic sublayers (Chandki et al., 2011). The biofilm matrix is comprised of microbial consortia with indwelling water channels, assorted cells and extracellular polymers that are composed of glycoproteins, polysaccharides, and proteins (Christensen, 1989; Christensen and Characklis, 1990). The primary colonizers form a biofilm by auto-aggregation (attraction between same species) and co-aggregation (attraction between different species). The attached bacteria multiply and secrete an extracellular matrix, which results in a mature mixed-population biofilm (Chandki et al., 2011).

Genetic adaptation is an important mechanism of survival, which results from genetic mutations and recombination, regulation of expression of the existing genetic material, and acquisition of genetic material. The genomic plasticity or metabolic flexibility of expression within bacterial cells helps them survive rapidly changing environmental conditions and to live in diverse environmental niches (Brooks et al., 2011). Bacterial cells can colonize various parts of the human body by modifying their regulatory and metabolic activities (Yang et al., 2016). Various pathogenic bacteria possess the ability to move from the external environment to the human body by changing the nutrient uptake mechanism and the ability to resist primary and secondary immune defenses (Pickard et al., 2017). Bacterial cells can also alter their gene expression and convert from the planktonic form to the sessile form by enclosing themselves within extracellular polymeric substances (EPS) (Berlanga and Guerrero, 2016). Recent studies in the field of biofilm have focused predominantly on molecular genetics that regulate the formation of biofilms by conversion of planktonic cells to sessile forms (Davey and O’toole, 2000).

Biofilms can resist various types of antimicrobial agents (Lewis, 2001). Although more research is needed to understand the molecular mechanism behind the formation of biofilm, it is known that numerous genes change the metabolomics of bacterial cells, leading to their conversion from planktonic to sessile forms. Biofilms formed on the surface of medical devices include Gram-positive as well as Gram-negative cells. The most common Gram-positive bacteria are *Enterococcus faecalis, Streptococcus pyogenes, Staphylococcus mutans, Staphylococcus epidermidis, Bacillus subtilis*, and *Staphylococcus aureus*, whereas Gram-negative bacterial cells are *Klebsiella pneumoniae, Escherichia coli, Proteus mirabilis*, and *Pseudomonas aeruginosa* (Kwakman et al., 2006). Apart from bacteria, various groups of filamentous fungi and yeasts can form biofilms on abiotic surfaces, but these biofilms differ from those formed by bacterial cells: In yeast, the attachment is mediated by special types of proteins known as adhesion proteins (Willaert, 2018), which are usually located outside the cell wall and are subjected to epigenetic switching that results in the development of stochastic expression pattern (Verstrepen and Klis, 2006). Biofilm-derived *L. pneumophila* replicate more in murine macrophages than in planktonic bacteria. The biofilm is the most important determinant of survival and proliferation of bacteria in warm, humid environments.

To remove biofilms from medical devices, coatings made of acylase and α-amylase are used (Ivanova et al., 2015). The enzymes amylase, cellulase, protease, DNase, alginate, and lyase are reported to support removal of biofilms from medical devices (Stiefel et al., 2016). Therefore, enzymes can be considered natural agents for degradation of biofilm.

## EPS: The Most Crucial Component of Biofilm Matrix and the Main Target for Antibiofilm Agents

**Figure 2:**
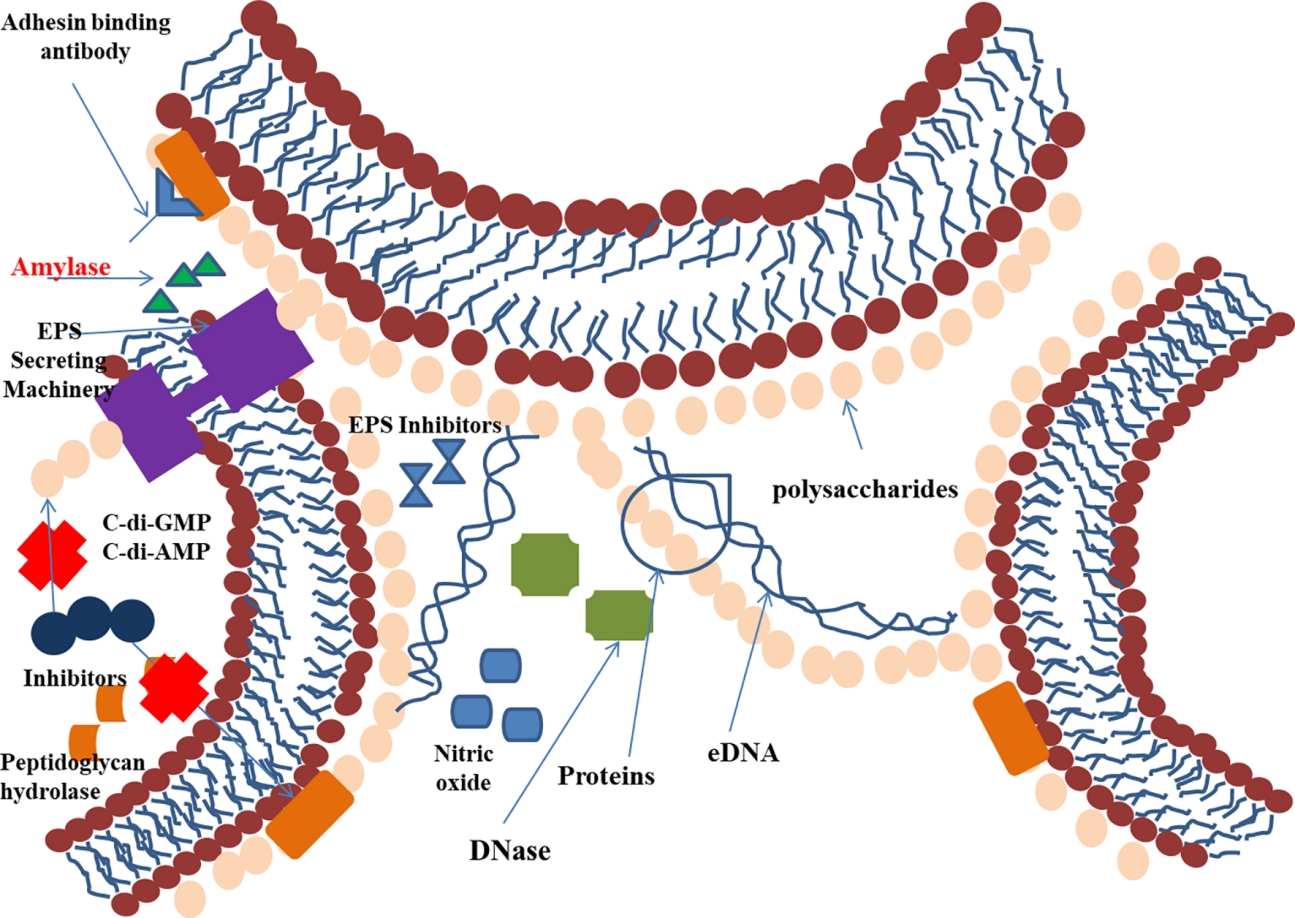
EPS as a site of action for antibiofilm agents.

The composition of the EPS matrix greatly varies structurally and temporally, based on the type of microorganism, availability of substrate, local mechanical shear force, and the environment of the host. The EPS matrix helps in cell-cell adhesion, adhesion to the surface, and aggregation (Flemming and Wingender, 2010), whereas the 3D scaffold helps protect the sessile communities from antimicrobial therapies and provides mechanical stability. EPSs also can reorient the chemical and nutrient gradient and delineate the pathogenic environment; thus, they are important for determining virulence (Hobley et al., 2015; Flemming et al., 2016). This feature makes EPS an important target for therapeutics that act by disaggregating bacterial cells wall to slow the growth of pathogenic bacteria (Gunn et al., 2016). The EPS can be targeted by inhibiting its production or preventing its binding or adhesion to surfaces, thus interfering with biofilm development (**Figure 2**).

## Enzymes as Antibiofilm Agents

The enzymes that aid in removing existing biofilms can be categorized into six major groups: transferases, oxidoreductases, hydrolases, lyases, and ligases or synthetases (**Table 2**). The enzyme-associated antifouling activity involves the lysis of cells by degrading cell-membrane components and destabilizing its anchoring to the solid surface. Saccharolytic enzymes produced by certain bacterial cells disintegrate the biofilm, resulting in release of the cells (Gupta et al., 2016). The enzymes prevent the production of adhesives and the formation of EPS, thus preventing the formation of biofilm (Oulahal et al., 2007). Starch is a predominant chemical component in the formation of biofilms (Klein et al., 2009); thus, enzymatic degradation of the polysaccharide results in the removal of the biofilm.

**Table 2:** Role of enzymes as antibiofilm agent on abiotic surfaces.

Combinatorial Therapeutics	Surface	Species responsible for the formation of biofilm	Extent of biofilm reduction	References
α-chymotrypsin+α-amylase+ lipase	Glass plate	*Pseudoalteromonas* and *Rhodobacter* sp.	90% reduction	(Zanaroli et al., 2011)
Quatro Zyme R (lipase, protease, cellulase, amylase) and Reflux R E2001 (protease and lipase)	Ultrafiltration membranes of dairy industry	*Klebsiella oxytoca*	6.02 and 6.15 log CFU/cm^2^ reduction	(Tang et al., 2010)
		Dual biofilm formed by *Klebsiella oxytoca*	5.31 log CFU/cm^2^ reduction	
α-Amylase+ β-Glucuronidase+ Glucoseoxidase+ Dextranase+Protease +Pectinase	Stainless steel	Multispecies biofilm formation by *Lactobacillus brevis*, *Leuconostoc mesenteroides* and *Saccharomyces cereveceae*	Single dose of enzymes for 2 log cycles	(Walker et al., 2007)
Anionic Buffer +α-amylase	Stainless steel	*Bacillus mycoides*	2.89 log CFU/cm^2^ reduction	(Lequette et al., 2010)
Amyloglucosidase+Ultrasound	Stainless steel	*Pseudomonas aeruginosa*	90% removal	(Oulahal et al., 2007)
Amyloglucosidase+EDTA+Ultrasound	Stainless Steel	*S. aureus*	100% removal	(Oulahal et al., 2007)
Dextranase	Teeth	*S. mutans*	89.34% removal	(Ren et al., 2018)
Mutanases	Teeth	*S. mutans*	75% removal	(Ansari et al., 2017)

## Amylases

Amylase is an important group of enzymes, which are classified into α, β, γ subtypes, isoamylase, glucoamylase, and others. α and β-amylase have the potential to catalyze the hydrolysis of chitosan (Rokhati et al., 2013) and reduce its molecular weight, which makes it more soluble (Pati et al., 2020a) and thus may lead to diversified applications (Pati et al., 2020b). Since the discovery of the first amylase by Anselme Payen, in 1833 (Krikorian, 1970), many more have been found within living systems that have specific substrates (Guzmàn-Maldonado et al., 1995; Gupta et al., 2003). Amylases can be found in both plant and microbial sources. Based on the mode of action, amylases can be classified into exo-amylases and endo-amylases. Exo-amylases hydrolyze substrates from the non-reducing ends, resulting in shorter end products (Gupta et al., 2003), whereas endo-amylases act on internal glycosidic linkages in a random manner within starch molecules, resulting in oligosaccharides of various lengths (Stütz and Wrodnigg, 2011). Multiple amylases present in *L. pneumophila* are essential for hydrolyzing polysaccharides into glucose and in helping intracellular proliferation. Amylase also helps trigger pro-inflammatory responses, which further helps prevent bacterial replication (Douglas et al., 1990, Murray et al., 1992, Souza et al., 2020).

### α- Amylases: Structural and Functional Characteristics

α-1,4-glucan-4-glucanohydrolase, EC. 3.2.1.1, which predominantly acts on starch (polysaccharide) as the major substrate, consists of two glucose polymers – amylose and amylopectin. α-amylase helps in the hydrolysis of α-1,4 and α-1,6-glycosidic linkages, which results in the formation of small glucose (monosaccharides) and maltose (disaccharide). α-amylase is essentially a metalloenzyme, which requires metals such Ca^2+^, for maintaining the stability of the enzyme molecule (Saboury, 2002). Sequence alignment studies have found that α-amylases possess four conserved regions that are also present within the β strands (Møller et al., 2004). The α-amylases are present widely within plants, microorganisms, and higher animals (Kandra, 2003). The end products obtained by the action of this amylase are oligosaccharides of various length of limit dextrin and configurations (Van Der Maarel et al., 2002). The end products also consist of the of branched malto- oligosaccharides possessing 6-8 glucose units that have -1,6 and -1,4, linkages, maltose, and maltotriose (Whitcomb and Lowe, 2007). These amylase enzymes can bind with substrates *via* catalytic groups that catalyze breakage of the glycosidic bond (Iulek et al., 2000).

### β -Amylases: Structural and Functional Characteristics

β-amylase (E.C.3.2.1.2, α-1,4-D-maltoglucan hydrolase) can hydrolyze starch to β-maltose and β-limit dextrin (Chia et al., 2004). Most of the commercial amylases are obtained from plant sources, but microbial sources are preferred because of lower cost of production, greater stability, easy genetic manipulation, and easier extraction (Ray and Nanda, 1996). Also, fungi have become a source of β-amylases (Ray, 2004).

### Glucoamylases: Structural and Functional Characteristics

Glucoamylase (EC 3.2.1.3) successively cleaves each glycosidic starch bond from the non-reducing end to form glucose. α-glucosidase (EC 3.2.1.20) resembles glucoamylase when the α-1,4-linkages are hydrolyzed from the non-reducing ends of alpha-glucans. However, the two enzymes adopt numerous pathways of distinct anomeric arrangements to release glucose. Glucoamylase inverts the α-d-glucose release mechanism, while alpha-glucosidase follows the retention process to generate α-d-glucose (Kumar and Satyanarayana, 2009). Most glucoamylases are multidomain enzymes that consist of a catalytic domain linked by an O-glycosylated linker region to a starch-binding domain (Sauer et al., 2000). A glucoamylase [*gamA*] gene encodes a eukaryotic-like glucoamylase that is responsible for the degradation of glycogen and starch in bacteria such as *Legionella pneumophila*.

### Human Salivary and Pancreatic Amylases: Structural and Functional Characteristics

**Figure 3:**
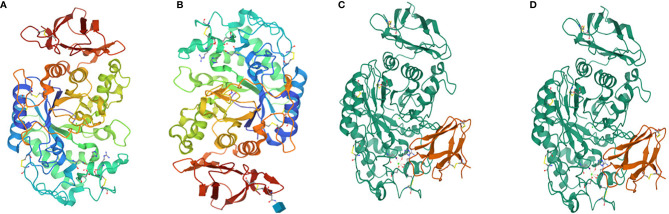
**(A)** Human salivary alpha amylase, **(B)** human pancreatic amylase **(C)** alpha amylase, and **(D)** beta amylase.

Salivary amylase is a glucose-polymer enzyme, which cleaves large starch molecules into dextrin and subsequently into smaller malto-oligosaccharides containing α-D-(1,4) linkages, iso-malto-oligosaccharides containing α-D-(1,6) linkages, the trisaccharide maltotriose, and the disaccharide maltose (Jacobsen et al., 1972). Salivary and pancreatic amylases hydrolyze starch (Bonnefond et al., 2017). Human pancreatic amylase cannot cleave the 1,6-linkages nor the terminal glucose residues (Whitcomb and Lowe, 2007). Human amylase is a calcium-containing enzyme comprised of 512 amino acids with a single chain of oligosaccharide having a molecular weight of 57.6 kDa (Whitcomb and Lowe, 2007). The protein is comprised of three domains, namely, A, B and C, of which A is the largest and is mainly barrel shaped with eight superstructures. The B domain is located between A and C and is linked with A *via* disulphide bonds. The C domain has a sheet-like structure that remains attached to the A domain *via* a simple polypeptide chain, which appears as an independent domain having no known function. The active site of the amylase is between the carboxyl end of the A and B domains that have the calcium ion and help stabilize the three-dimensional structure (Muralikrishna and Nirmala, 2005) (**Figure 3**).

### Amylase-Associated Biofilm Removal

The oral cavity contains biofilms of microbial species such as *C. albicans, C. glabrata, E. faecalis, S. mutans, V. dispar* and *F. nucleatum* (Berger et al., 2018). Though saliva is rich in amylase, plaque formation has been reported to occur in the presence of the enzyme. Natural selection has dictated the mechanisms working *in vivo* (not often mimicked *in vitro*). Amylase has potent antibiofilm activity (Kalpana et al., 2012). However, *in vitro* studies have shown that α-amylase is a potential antibiofilm agent against biofilm-forming bacterial species such as *S. aureus* and *P. aeruginosa* (Lahiri et al., 2021a). Although α-amylase did not have much effect on the biofilm formed by *S. epidermidis*, it reduced biofilm formation, and it completely inhibited biofilm formation by *S. aureus* (Bradford, 2011). A 79% reduction in the biofilm was observed in *S. aureus* when challenged with enzyme for 5 minutes. Increase in the concentration of amylase from 10 mg/mL to 100 mg/mL decreased the biofilm formation from 72% to 90% and inhibited EPS by 82% (Bradford, 2011). Six strains of MRSA had a dose-dependent response to α-amylase of about 92%–97% reduction in biofilm biomass, which is evidence that α-amylase is a potent inhibitor of biofilm formation (Watters et al., 2016).

In a study by Kalpana et al. (2012), the α-amylase obtained from *Bacillus subtilis* had antibiofilm activity against *S. aureus* (MRSA), *P. aeruginosa*, and *V. cholerae* (Kalpana et al., 2012). The crude enzyme also was effective against *S. aureus* and *P. aeruginosa* and degraded the EPS, with efficacy of 51.8% to 73.1%; the purified enzyme reduced biofilm formation by 43.8% to 61.7%. Stronger antibiofilm effect was found in the work of Watters et al. (2016), where human plasma (10%) was supplemented in a culture of *S. aureus* biofilm; α-amylase was effective against both methicillin-sensitive and methicillin-resistant organisms. The work of Molobela et al. (2010) showed the successful use of α-amylase from *Bacillus amyloliquefaciens* and glucoamylase from *Aspergillus niger* on the Gram-negative biofilm-forming bacteria *Pseudomonas fluorescence.* EPS was reduced by 42.5% in the presence of the enzyme in a challenge of 90 minutes. Microscopic studies were performed to assess the reduction of biofilm and the ability of the enzyme to degrade the EPS and disperse the cells, which resulted in reduction of the biofilm (Molobela et al., 2010). Amylase (glucoamylase and amyloglucosidase units) was successfully used as a hydrolytic enzyme in controlling coaggregation in dental plaque, though it did not significantly alter bacterial viability within the plaque microcosm. (Ledder et al., 2009). Enzymatic inhibition of polysaccharides has been investigated, and α-amylase was found the most efficient enzyme (Divakaran et al., 2011).

The α-amylase produced from *A. oryzae* inhibits the biofilm formed by *S. aureus*. β-amylase is an exo-acting carbohydrolase that hydrolyzes the α-1,4-glucosidic linkages of starch only from the non-reducing end of the polysaccharide. The α-amylase can act anywhere on the substrate, thus it tends to act faster than does β-amylase (Toda et al., 1993). The enzyme prevents the surface adherence and helps in the dispersal of the cells, for instance the biofilm formed by *Aggregatibacter actinomycetemcomitans* when treated with dispersin B and poly-β-1,6-N-acetyl-D-glucoseamine hydrolyzing enzymes (Kaplan et al., 2003a; (Kaplan et al., 2003b; Izano et al., 2008). It has often been observed that a single enzyme is not sufficient to reduce biofilm formation. Therefore, researchers often test combined treatments of biofilms with various enzymes. The combination of levan hydrolase, amylase, and dextrin hydrolase has helped remove the biofilm on inanimate objects (Hatanaka and Sugiura, 1993), and beta-glucanase, protease, and alpha amylase in combination were effective in removing industrial slime (Wiatr, 1990).

The activity of β-amylases in inhibiting the biofilm is less than that of α-amylases. The reason for this difference is that β-amylases can be an exo-acting carbohydrase, which can hydrolyze 1,4-glucosidic linkages of the starch from the non-reducing end. This action opposes the activity of the α-amylases, which can act faster at any position on the substrate (Toda et al., 1993). Although amylases have acclaimed biofilm degrading activity, few reports are available on the biofilm-inducing activity of amylase.

## Salivary Amylase-Associated Biofilm Formation

α-amylase is the most abundant enzyme produced primarily from the serous cells of the parotid, submaxillary, sublingual, and minor glands. The reported concentration of amylase in the saliva ranges from 0.04-0.4 mg/ml and comprises about 5% of the total salivary proteins (Jacobsen et al., 1972). The concentration of α-amylase increases with the intake of food (Rohleder et al., 2006). Inui et al. (2019) found that the stimulatory protein responsible for the development of biofilm by *Streptococcus anginosus* and *Streptococcus gordonii* was enhanced in the presence of saliva (Inui et al., 2019). Salivary-α-amylase belongs to the family of α-1,4-glucan-4-glucanhydrolase, which catalyzes the α-1,4-glycosidic bonds of glycogen, starch, and other polysaccharides (Scannapieco et al., 1989; Scannapieco et al., 1992). Digestion of starch involves enzymatic degradation, beginning in the oral cavity with the formation of maltose and maltodextrin. The result is an abundance of carbohydrate for nutrition of the oral bacteria. Apart from having hydrolyzing activity, α-amylase can be adsorbed onto the tooth enamel (Al-Hashimi and Levine, 1989; Dufour et al., 2014), where it is a substrate for bacteria (Brown et al., 1999). The prominence of α-amylase in the saliva and the dental pellicle (Jensen et al., 1992; Yao et al., 2001) is a potent precursor for the development of dental biofilm. α-amylase can convert the long chains of malto-oligosaccharides to maltose as their end product, and glycosylated α-amylase is a potent converter of maltotriose into maltose and glucose (Koyama et al., 2000). The amylase-binding site is present in the glycosylated and the non-glycosylated forms of the enzyme (Scannapieco et al., 1989). Salivary α-amylase exists as monomeric (Ragunath et al., 2008) and dimeric (Fisher et al., 2006) forms that possess calcium and chloride ions, which enhance its enzymatic activity. The ability of α-amylase to bind with the bacteria is a calcium-independent mechanism that is independent of hydrolysis. The active site of the enzyme enables the binding of the saccharide hydrolysate of the starch. The enzyme also has several oligosaccharide binding sites, which enhance affinity of α-amylase to the starch granules (Ragunath et al., 2008). The secondary oligosaccharide binding sites are important sites for the bacteria (Ragunath et al., 2008; Spöring et al., 2018). Mutations at the aromatic rings on the secondary oligosaccharide residues decrease the affinity of *Streptococcus gordonii* for the α-amylases (Ragunath et al., 2008). The α-amylase retains its enzymatic activity even though being attached to the bacterial cells; thus, the site for enzymatic activity needs to be distinct from the bacterial binding site (Scannapieco et al., 1989) (**Table 3**).

**Table 3:** Streptococcal proteins interacting with salivary amylase.

Streptococus species	α -Amylase binding component	Interaction between bacterial surface and α -amylase	Binding of bacteria to surface bound α -amylase	References
*S. australis*	AbpA-like, AbpB- like, novel protein	Unknown	Unknown	Nikitkova et al., 2013; Haase et al., 2017
*S. cristatus*	AbpA-like, AbpB- like, novel protein	Unknown	Unknown	Gwynn and Douglas, 1994; Haase et al., 2017
*S. gordonii*	AbpA, AbpB	Positive	Confirmed	Scannapieco et al., 1992; Gwynn and Douglas, 1994; Rogers et al., 2001
*S. infantis*	AbpA-like, novel protein	Unknown	Unknown	Nikitkova et al., 2013; Haase et al., 2017
*S. mitis*	AbpC, novel proteins	Unknown	Unknown	Brown et al., 1999; Vorrasi et al., 2010; Haase et al., 2017
*S. mutans*	Pili	Unknown	Unknown	Ray et al., 1999
*S. oralis*	AbpA-like, novel protein	Unknown	Unknown	Haase et al., 2017
*S. parasanguinis*	AbpA, AbpB	Positive	Unknown	Gwynn and Douglas, 1994; Brown et al., 1999; Liang et al., 2016; Haase et al., 2017
*S. salivarius*	AbpA-like	Unknown	Unknown	Haase et al., 2017
*S. sanguinis*	Pili	Unknown	Unknown	Okahashi et al., 2011
*S. vestibularis*	AbpA	Unknown	Unknown	

**Figure 4:**
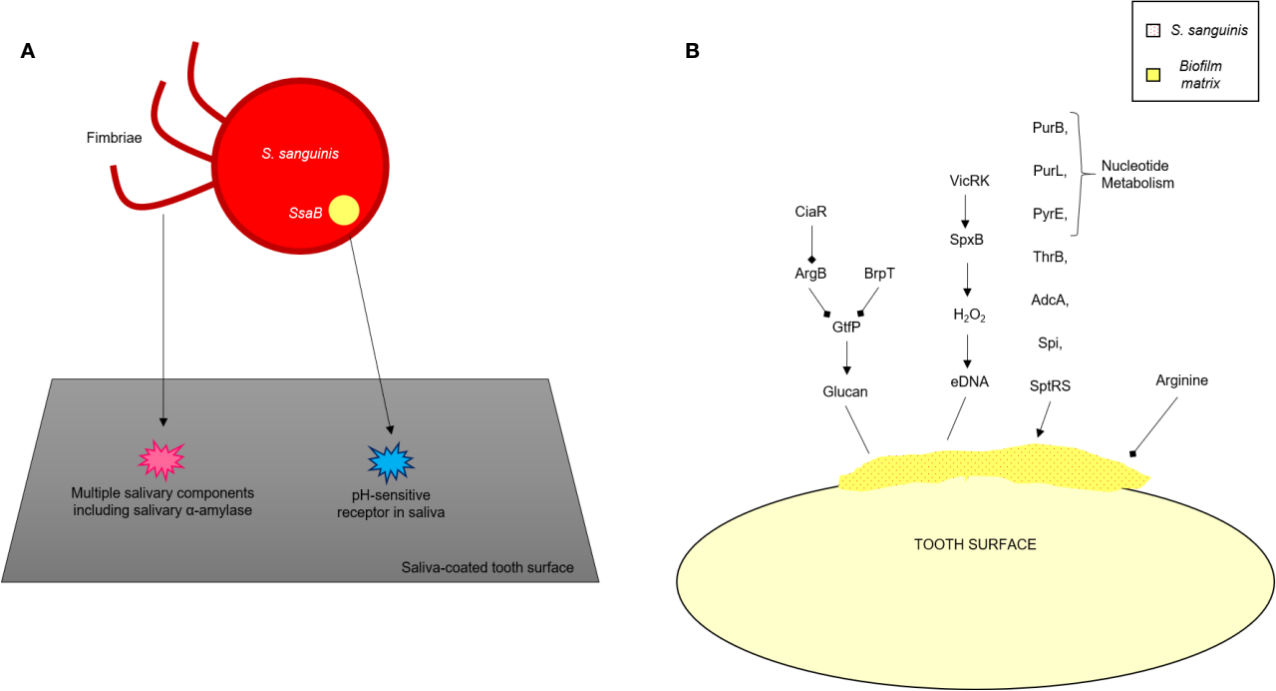
Mechanism of formation of biofilm by *S. gordonii*.

**Figure 5:**
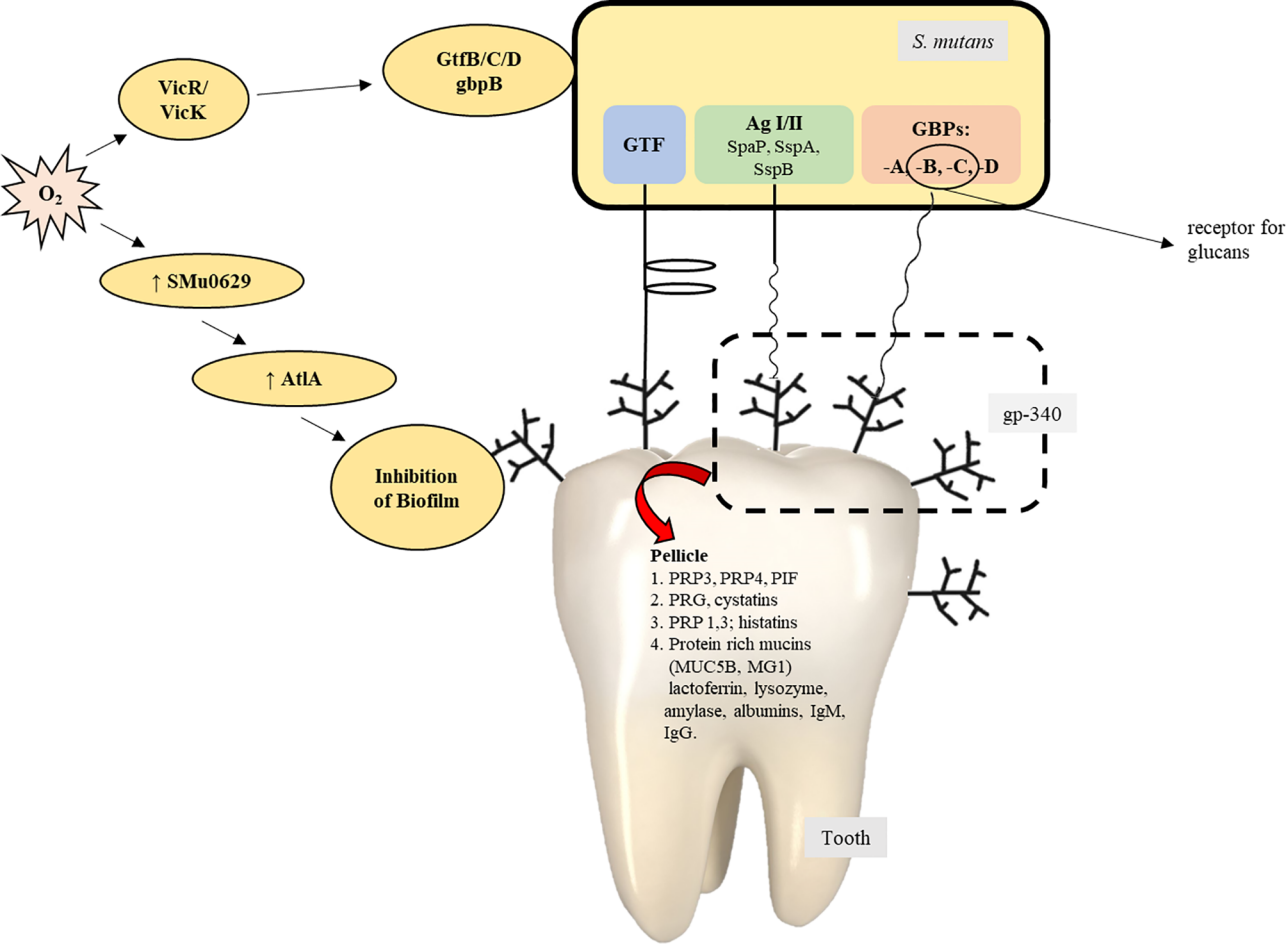
The contribution/role of *Streptococcus mutans* in the process of the formation of biofilms (Zhu et al., 2018).

The bacterial surface adhesins, under the influence of salivary agglutinins, help form biofilm (Ahn et al., 2008; Khan et al., 2011). Salivary amylase plays a vital role in the formation of the *S. mutans* biofilm (Ahn et al., 2008; Klein et al., 2010; Khan et al., 2011). Some conditions result in the expression of virulence factors within *S. mutans* and favor the development of the oral biofilm: the presence of other organisms and their interaction with *S. mutans* (Wen et al., 2010), ability of the organisms to survive in aerobic conditions (Ahn et al., 2008), and the availability of oxygen, which is responsible for bringing about variations in the composition of bacterial cell surfaces by the production of autolysins, and activation of the signal transduction system of VicRK. The AtlA autolysin is controlled by the SMu629 gene expression of oxidoreductases. Anaerobic conditions inhibit the expression of genes that control the overproduction of AtlA autolysins, thus inhibiting the formation of the biofilm (**Figure 4**). Pilus biogenesis gene PilC can be bound to salivary α-amylase by its multiple salivary components (**Figure 5**).

### Amylase-Binding Proteins

Amylase-binding *Streptococci* (ABS) express different proteins ranging from 20-87 kDa which bind salivary a-amylase *in vitro* (Gwynn and Douglas, 1994; Brown et al., 1999; Haase et al., 2017). The heterogeneity of these proteins varies from species to species with some expressing more than one ABP. It has been observed that lower molecular weight ABPs range from 20-36 kDa, whereas higher molecular weight ABPs range from 82-87 kDa. BLAST searches terminal sequences obtained from several ABPs identified AbpA, AbpB, AbpC and several unique ABPs in the NCBI database (Haase et al., 2017). The most predominantly studied ABPs are AbpA (20 kDa) and AbpB (82 kDa) from *S. gordonii* (Chaudhuri et al., 2008). Amylase-binding protein C (AbpC) obtained from *S. mitis* which is about 36 kDa was cloned and sequenced.

Although it is able to bind salivary a-amylase, sequence analysis showed no homology to AbpA or AbpB (Vorrasi et al., 2010). Further in silico analysis suggested homology with choline-binding proteins (Haase et al., 2017). Alignment and phylogenetic analysis found that ABPs cluster into at least six phylogenetic groups with no evidence that one group evolved from another (Haase et al., 2017).

### Amylase-Binding Protein A

AbpA is the most studied ABP. Obtained from *S. gordonii*, it is about 20kDa and is an externally located and cell wall-associated target protein that is expressed maximally at the mid-log phase of bacterial growth (Brown et al., 1999). Abp A is an essential receptor for binding of α-amylase; inactivation of it eliminates the α-amylase binding capacity of the bacterium (Rogers et al., 2001). Abp A is located on the surface of the cells, as revealed by immunogold electron microscopy (Scannapieco et al., 1992). Cells in the logarithmic phase can bind α-amylase better than can those in the stationary phase; this feature predicts that the receptor is mainly present in the nascent cell wall and is shed into the supernatant as the cell matures. The electron microscopic studies also indicated that binding of the α-amylase does not change the morphology of the bacterial cells or perturb the cell surface (Scannapieco et al., 1992). Studies of the biofilm-forming genes of *S. gordonii* by Tn 916 mutagenesis revealed that *abpA* is the potential biofilm-forming gene (Loo et al., 2000; Costa et al., 2020). Other research revealed that the absence of *AbpA* in *S. gordonii* impaired biofilm formation on saliva coated flow cells (Rogers et al., 2001). *S. gordonii* and other ABS are important in the formation of oral biofilm by metabolism of the dietary starch and delivery of nutrients to the non-ABS species in the biofilm. Thus, this type of interaction makes ABS a competitor to the pathogenic species of bacterial cells. Studies performed with the *abpA* mutant strain, with the help of pathogen-free Osborne-Mendel rats, yielded results contradictory to those of the *in vitro* studies. The *abpA* mutant strains reportedly colonize on the tooth surface better than do the wild type, especially when the rats are provided a starch diet (Tanzer et al., 2003). The expression of the glucosyltransferase G, which is one of the potent enzymes promoting the formation of biofilm, was found greater in the *abpA* mutant strain (Tanzer et al., 2003). Chaudhuri et al. (2007) have reported that glucosyltransferase G forms a complex with AbpA and salivary amylase to form biofilm by *S. gordonii*. The complex enhances the enzymatic activity of glucosyltransferase G and salivary amylase (Chaudhuri et al., 2007) and the activity of amylase in *S. mutans*.

### Amylase-Binding Protein B

AbpB, with a molecular weight of 82 kDa protein, is co-precipitated with α-amylase, AbpA, and glucosyltransferase G. Although AbpB has the ability to bind α-amylase, as confirmed by Western blot studies (Chaudhuri et al., 2008), it does not have homology with AbpA or AbpC. AbpB, however, has homology with bacterial peptidases (Chaudhuri et al., 2008). Abp B shows predominance in hydrolytic activity for Ala-Pro, Gly-Prp and Arg-Pro peptides, which suggests that it restricts enzymatic activities to protein-containing proline-containing residues (Chaudhuri et al., 2008). AbpB has been found to play an important role in the colonization of bacterial cells within the oral cavity, which helps in nutrient acquisition by various pathways. It also helps the bacterial cells present in the oral cavity and dental plaques obtain nutrients from the salivary-proline rich proteins (Chaudhuri et al., 2008).

### Amylase-Binding Protein C

AbpC is a 36 kDa protein consisting of 292 amino acid residues, with a hydrophobic signal peptide comprising the first 31 N-terminal amino acid residues, as obtained from the supernatant of *S. mitis* (Vorrasi et al., 2010). AbpA protein does not share homology with AbpA and AbpB but has similarities at the level of amino acids. AbpC is associated with the bacterial cell wall, and it also is a potent receptor of α-amylase.

## Conclusion

Biofilm, being the consortia of microbial species and mostly responsible in the development of chronic human diseases, is an important target for therapeutics, as most antimicrobial agents cannot penetrate the EPS matrix of the biofilm (Campoccia et al., 2006). EPS degrading agents – more precisely, natural agents – are being prioritized to manage biofilms. Amylases, which hydrolyze the polysaccharide backbone of EPS, may be useful in the management of biofilms. A combination of enzymes may be used to reduce the accumulation of biofilm on various biotic and abiotic surfaces (Stiefel et al., 2016); the combination would contain amylase, especially α-amylase, β amylase, and amyloglucosidase. High levels of α-amylase, as present in saliva, may enhance or control biofilm formation on dental surfaces with the help of proteins such as AbpA-binding protein. *Streptococcus mutans*, (which does not bind amylase) builds a potentially more cariogenic biofilm when sucrose is combined with starch because starch hydrolysates may be acceptors during glucan synthesis, altering the branching and the tridimensional structure. Starch by itself is not a “molecular backbone” for those biofilms, but it could enhance their pathogenicity (Klein et al., 2009). Amylase, in combination with other enzymes, may have antibiofilm efficacy against pathogens such as *E. coli, S. aureus*, and methicillin-resistant *Staphylococcus aureus.* Hence, the paradoxical actions of amylase raise questions about the exact role of amylase on biofilm *in vivo*.

## Author Contributions

All authors contributed to the article and approved the submitted version.

## Funding

This work was supported by Fundamental Research Grant Scheme (304/PPSK/615059) awarded by Ministry of Higher Education, Malaysia and Odisha Higher Education Program for Excellence and Equity (OHEPEE), Govt of Odisha.

## Conflict of Interest

The authors declare that the research was conducted in the absence of any commercial or financial relationships that could be construed as a potential conflict of interest.
